# Use of Geographically Weighted Poisson Regression to examine the effect of distance on Tuberculosis incidence: A case study in Nam Dinh, Vietnam

**DOI:** 10.1371/journal.pone.0207068

**Published:** 2018-11-12

**Authors:** Long Viet Bui, Zohar Mor, Daniel Chemtob, Son Thai Ha, Hagai Levine

**Affiliations:** 1 Center for Research–Consulting and Support of Community Health, Ha Noi, Vietnam; 2 Braun School of Public Health and Community Medicine, Hebrew University—Hadassah, Jerusalem, Israel; 3 Sackler Faculty of Medicine, Tel-Aviv University, Tel-Aviv, Israelg; 4 Tel Aviv Department of Health, Tel Aviv, Israel; 5 Department of Tuberculosis and AIDS, Ministry of Health, Jerusalem, Israel; 6 Administration of Medical Services, Ministry of Health of Vietnam, Ha Noi, Vietnam; University of Essex, UNITED KINGDOM

## Abstract

**Objectives:**

This study aimed to examine the potential of combining routine tuberculosis (TB) surveillance and demographic and socioeconomic variables into the Geographic Information System (GIS) to describe the geographical distribution of TB notified incidence in relation to distances to health services as well as local demographic and socioeconomic factors, including population density, urban/rural status, and household poverty rates in Nam Dinh, Vietnam. It also aimed to compare the conventional Generalized Linear Models (GLM) Poisson regression model and Geographically Weighted Poisson Regression (GWPR) models in order to determine the best fitting model that can be used to investigate the relationship between TB notified incidence and distances and the social risk factors.

**Methods:**

The data of new and relapse patients with all forms of TB aged ≥15 years residing in Nam Dinh (Vietnam) from 2012 to 2015 were collected from the Administration of Medical Services’ (Ministry of Health of Vietnam) TB surveillance database. Data on the population and household poverty rates from 2012 to 2015 were gathered from the Nam Dinh Statistical Office. Distances between communes and the nearest TB diagnostic facilities in districts were computed. The TB notified incidence per 100,000 population was denoted by indirect age and sex standardized incidence ratio. GLM Poisson regression and GWPR were performed to assess the relationship between distance and TB incidence.

**Results:**

The average notified TB incidence level measured from 2012 to 2015 is 82 per 100,000 population (range: 79-84/100,000). The distance to the nearest TB diagnosis presents a negative effect on TB notified incidence. By capturing spatial heterogeneity, the GWPR may be better at fitting data (corrected Aikake information criterion [AICc] = 245.71, residual deviance = 221.12) than the traditional GLM (AICc = 251.53, residual deviance = 241.21)

**Conclusions:**

GIS technologies benefit TB surveillance system. Distances should be considered when planning methods of improving access for those who live far from TB diagnostic services, thereby improving TB detection. Additional studies must confirm the association between geographic distance and TB case detection and must explore other factors that may affect TB notified incidence.

## Introduction

Vietnam is among the 20 countries with the highest incidence of tuberculosis (TB) worldwide [1]. The Vietnam National Tuberculosis Programme (NTP) was established in 1986 based on principles which include Direct Observed Treatment and Short Course (DOTS) as recommended by the World Health Organization (WHO) [2,3]. The NTP is divided into four levels: central, provincial, district, and communal levels. At the central level, the National Lung Hospital is responsible for the overall implementation and supervision of the program. The provincial level covers regional lung hospitals and TB and lung disease departments of general hospitals. The district level oversees the provision of DOTS and follow-up treatment to patients. The communal level covers community volunteers who support the district in case detection, DOTS completion, and patient outreach to those are lost to follow-up. The Vietnam NTP currently covers nearly all 708 districts and 11,162 communes (compared with 40% and 18% in 1986, respectively) [2,4].

The NTP has made efforts to reach the Millennium Development Goals by reducing TB prevalence, incidence, and mortality by 4.6%, 2.6%, and 4.4%, respectively, every year from 1990 to 2013 [5]. Consequently, the incidence of TB declined from 375 to 209 cases per 100,000 population between 2000 and 2014 [6]. However, TB prevalence for Vietnam is likely greater than the published figures. A national prevalence survey conducted in 2007 showed that the TB prevalence in Vietnam is 1.6 times higher than that estimated by the WHO [7]. For this reason, better surveillance and community-driven approaches applied at the subnational level remain essential to achieve the arduous goal of depressing the TB prevalence rate in Vietnam to 20 per 100,000 the following.

Geographical Information System (GIS) is a computer-based system in which data that are linked to geographic space (known as spatial data) can be input, managed, processed, and retrieved. By considering “spatial-related aspects” (e.g., place, area or distance) it can provide intuitive information in the fields of epidemiology, communicable disease control, medical geography, environmental health, and health services planning in developing countries [8]. In the realm of TB, GIS has proven its advantages to DOTS strategy development and is increasingly being employed to identify the spatial patterns of TB observed at subnational levels and biological, social and demographic determinants [9–11].

Distance or proximity to a health facility is an important factor that affects the performance of health programs especially in developing countries [12]. The “distance decay effect”, a geographic term that reflects a significant decrease in the utilization of health services in correlation with increasing distance from residential locations to healthcare providers, has been described in various studies and should be taken into consideration when planning services or improving policies [13–16]. However, studies on the relationship between TB case detection and distances to TB diagnostic facilities are limited. A recent study conducted in Ethiopia found that longer distances to TB services are associated with lower TB notification rates [17].

Traditional global regression methods such as Ordinary Least Square and Generalized Linear Models (GLM) have been widely used in health studies [18,19]. However, such techniques disregard potential spatial variations in relationships between mortality and morbidity rates across space and therefore may generate biased results and conclusions. Local spatial variations can serve as meaningful information that can have implications for healthcare policy makers. From this issue, Geographically Weighted Regression (GWR) and Geographically Weighted Poisson Regression (GWPR) methods have been developed. GWR and GWPR are relatively new exploratory spatial data analysis techniques that incorporate non-stationary spatial structures of data into statistical models to generate local coefficients to elucidate spatial variations in relationships between dependent variables and covariates. GWPR is an extension of GWR and is used when dependent variables follow the Poisson distribution. GWPR was primarily developed for modelling mortality rates at small scales and is increasingly being used to examine associations between disease risks, incidence rates, mortality risks and spatially varying social factors [19–22].

In Vietnam, GIS has been rarely used in public health and epidemiological research. Given the potential uses of GIS for the TB program, this study was aimed to explore the potential of combining routine TB surveillance and demographic, as well as socioeconomic, variables into GIS (1) to describe the geographical distribution of TB notified incidence in relation to distances to health services and the local demographic and socio-economic factors such as population density, urban/rural status, and household poverty rates and (2) to compare the GLM Poisson regression and GWPR models to fit the relationship between TB notified incidence and distances and the given social risk factors.

## Methods

### Settings

The Nam Dinh province of Vietnam is located in the southern area of the Red River Delta, covering an area of 1,676 km^2^ and supporting a population of approximately 1.83 million. The province includes 10 districts with 35 urban and 194 rural communes [23].

The NTP in Nam Dinh is organized into 13 public facilities that deliver TB diagnostic services (S1A Fig), including 1 provincial lung hospital and 12 TB units housed in 12 district hospitals. The number of TB diagnostic facilities remained unchanged between 2012 and 2015. The provincial lung hospital employs 25 physicians while each TB unit employs 3 staff. The basic TB diagnostic services offered include smear sputum microscopy, tuberculin skin test, and chest X-ray. Sputum cultures areperformed at the provincial lung hospital. Xpert MTB/RIF assays have been available at the provincial lung hospital since 2015.

### Study design and data collection

We conducted a geographic epidemiological study based on the existing surveillance data. The dataset that contains the number of new and relapse patients with all forms of TB, notified in Nam Dinh between 2012 and 2015 and aged at least 15 years aggregated by age and sex at communal level was obtained from the TB surveillance database of the Administration of Medical Services (Ministry of Health of Vietnam). Two datasets, the first dataset that contains the names, administrative codes, and population stratified by age and sex and the second dataset that contains administrative codes, household poverty rates (measured from the proportion of household in the communes living below the national poverty line) of all communes from 2012 to 2015 were collected from the Nam Dinh Statistical Office.

A base map that covers the communal-level layer of Nam Dinh province was provided by the Vietnam National Remote Sensing Center and was projected to the World Geodetic System 84 Universal Transverse Mercator Zone 48N coordinate reference system [24]. This base map contains the name, administrative code, and land area of each commune.

We joined three datasets to the base map by administrative codes of communes using ArcGIS (ESRI Inc., Redlands, CA, USA, version 10.3). Then we calculated the annual population density of each commune from 2012 to 2015 by dividing the total population in the respective year by its land area.

### Statistical analysis

The notified incidence is measured as the number of new and relapse TB cases notified in a given year per 100,000 population. The confidence interval (CI) of notified incidence was obtained based on the assumption that the observed incident cases follow the Poisson distribution.

The TB indirect age and sex standardized incidence ratios (SIR) of commune i from 2012 to 2015 were calculated using the formula:

$$SIR=O_{i}/E_{i}$$

where O_i_ denotes the average number of TB cases notified in commune i during the study period and E_i_ as the expected number of notified TB cases for commune i and is calculated using the following formula:

$$E_{i}=\sum r_{sex,age}*n_{sex,age,i}$$

where sex ϵ (male, female), age ϵ (15–24 years, 25–34 years, 35–44 years, 45–54 years, 55–64 years, and 65+ years), r as the national sex-age reference TB incidence rates estimated from the national notification rates reported by the WHO from 2012 to 2015 [25], and n as the average mid-year population of each sex-age group of the commune i.

### Variable selection

Previous studies showed that higher TB incidence are associated with overcrowding, poverty and poor sanitation [26–28]. Several studies use local Gini index (representing the income distribution of residents) or income per capita and the Human Development Index to represent the socioeconomic exploratory variables [29,30]. Unfortunately, these indicators are not available in Vietnam at the provincial level. We therefore used household poverty rates as a proxy for the socioeconomic status of the communes.

Given that the observed and expected number of notified TB cases follows the Poisson distribution, the GLM can be written as follows:

$$ln(O_{i}+1)=ln(E_{i})+\beta_{0}+\beta_{1}(DEN)+\beta_{2}(POOR)+\beta_{3}(DOM)+\beta_{4}(DIST)+\varepsilon_{i}$$ (1)

where β_0_ is the global intercept and β_j_ *(j = 1*,*2*,*3*,*4)* are model parameters corresponding to exploratory variables. We added a constant of one to the average observed number of notified TB cases to mitigate problems related to counts of zero. DEN is the average population density measured for 1,000 inhabitants per square kilometer. POOR is the average household poverty rate measured as proportions of household living below the national poverty line. DOM is the urban/rural setting of communes (urban = 1, rural = 0). DIST is the Euclidean distance in kilometers, which is a straight line running from centroids of a commune to TB diagnostic facilities of the same district calculated using the Near tool in ArcGIS. *ε* is the error term of commune i.

When applying GWPR, parameters are functions of geographical location u_i_ = (u_xi_; u_yi_) where (u_xi_; u_yi_) denotes two-dimensional co-ordinates of commune centroids:

$$ln(O_{i}+1)=ln(E_{i})+\beta_{0}(u_{i})+\beta_{1}(u_{i})(DEN)+\beta_{2}(u_{i})(POOR)+\beta_{3}(u_{i})(DOM)+\beta_{4}(u_{i})(DIST)+\varepsilon_{i}$$ (2)

Parameters of regression models for each regression point are estimated based on nearby observations, for which data on closer communes have a greater effect on results than data for more distant communes. Geographic weights are identified from a kernel function such as the Gaussian or bi-square kernel with fixed or adaptive bandwidth. The Gaussian kernel continuously decreases from the centre of the kernel but never decreases to zero. The bi-square kernel has an explicit threshold that assigns a weight of zero to observations made outside of the bandwidth. If regression points are fairly regularly spaced in the study area then a fixed kernel is a suitable choice. In contrast, an adaptive kernel is appropriate when the regression points are irregularly positioned [18].

### Calibration of GWPR models

We started with a traditional GLM (Eq 1) with all parameters fixed. A residual deviance test was performed to assess the goodness of fit of the global GLM. Multicollinearity between independent variables was analyzed from the variance inflation factor (VIF), and variables with VIF>5 were considered to be collinear and were excluded from the model [31]. Variables with a p value of <0.05 were considered statistically significant.

We used the GWR4 software to calibrate the regression equation presented in Eq 2. The newest version of GWR4 provides the use of convenient algorithms for integrating both fixed and spatially varying independent variables in the GWPR model (semiparametric or mixed model). Adaptive Gaussian and adaptive bi-square kernels were used to estimate the parameters due to the inconsistent in the observed distribution of sample points. We used the L to G (local to global) variable selection option to find the optimal combination of fixed and geographically varying exploratory variables. A golden section search was opted to find the optimal bandwidth. The corrected Aikake information criterion (AICc) and residual deviance were used to measure the goodness of fit of the GWPR models and global GLM. A golden section search and L to G variable selection routine both rely on the minimization of the AICc to find the best fitting model. A model with a lower AICc of more than 3 denotes a better fit and is considered statisticallysignificant. Further information on GWPR model settings can be found in Nakaya et al. [19] and GWR for Windows [32].

### Testing for spatial autocorrelations of model residuals

Spatial autocorrelation occurs when data for one location correlates with data for other nearby locations through space. Moran’s I coefficient has been commonly used to assess spatial autocorrelation [33]. Moran’s I ranges from -1 (data are perfectly dispersed) to 1 (data are perfectly clustered). When Moran’s I reaches zero, there is no spatial autocorrelation. In the GLM Poisson regression, spatial autocorrelation is a commonly encountered issue when the model cannot adjust for existing spatial heterogeneity. In GWPR, after accounting for non-stationary effects, it is expected that the estimated errors of each observation should not be related to the surrounding observations [18]. In this study we employed global and local Moran’s I values to examine the spatial autocorrelations of observed the TB cases, and we used global Moran’s I to test the spatial autocorrelation of residuals of the GLM Poisson regression and GWPR models. Futher information on global and local Moran’s I values was described by Tango [33] and Anselin [34].

### Ethical considerations

The study design was approved by the Administration of Medical Services (Vietnam Ministry of Health) and the requirement for informed consent was waived due to the retrospective nature of the study.

## Results

### TB notified incidence in Nam Dinh province from 2012 to 2015

Between 2012 and 2015, 6,036 new and relapse patients with all forms of TB, including smear positive, negative, or extra-pulmonary were reported in the Nam Dinh province. In 2012, 1,488 cases were detected. The number of TB cases slightly dropped to 1,447 cases in 2013, increased to 1,535 cases, and finally peaked to 1,556 cases in 2015. The notified incidence per 100,000 population of all TB forms marginally increased from 79 to 84 during this period. The average value of TB notified incidence for this period was recorded as 82 cases per 100,000 population (95% CI: 78–86).

A summary of notified TB cases and SIR values of 229 communes is presented in Table 1 and S1A Fig. The absolute number of average notified TB cases within the study period varies across the study area from 0 to 17 (median 6, interquartile range [IQR]: 4). The SIR ranges from 0 to 5 with a median of 1.20 and an IQR of 0.6. Three communes consistently presented no TB cases over the 4-year period. Global Moran’s I statistic showed that the observed rates of TB incidence had positive autocorrelations or clustered patterns (Moran’s I = 0.56, p < 0.0001). The local Moran’s I value shown in S1B Fig showed local clusters of notified TB cases measured from certain TB diagnostic units.

**Table 1:** Summary of 4-year average TB notified incidence values for 229 communes.

	Average notified TB cases	Standardized Incidence Ratios (SIR)
**Min**	0	0
**1**^**st**^ **quartile**	4	0.90
**Median**	6	1.20
**3**^**rd**^ **quartile**	8	1.50
**Max**	17	5.0

**Fig 1:**
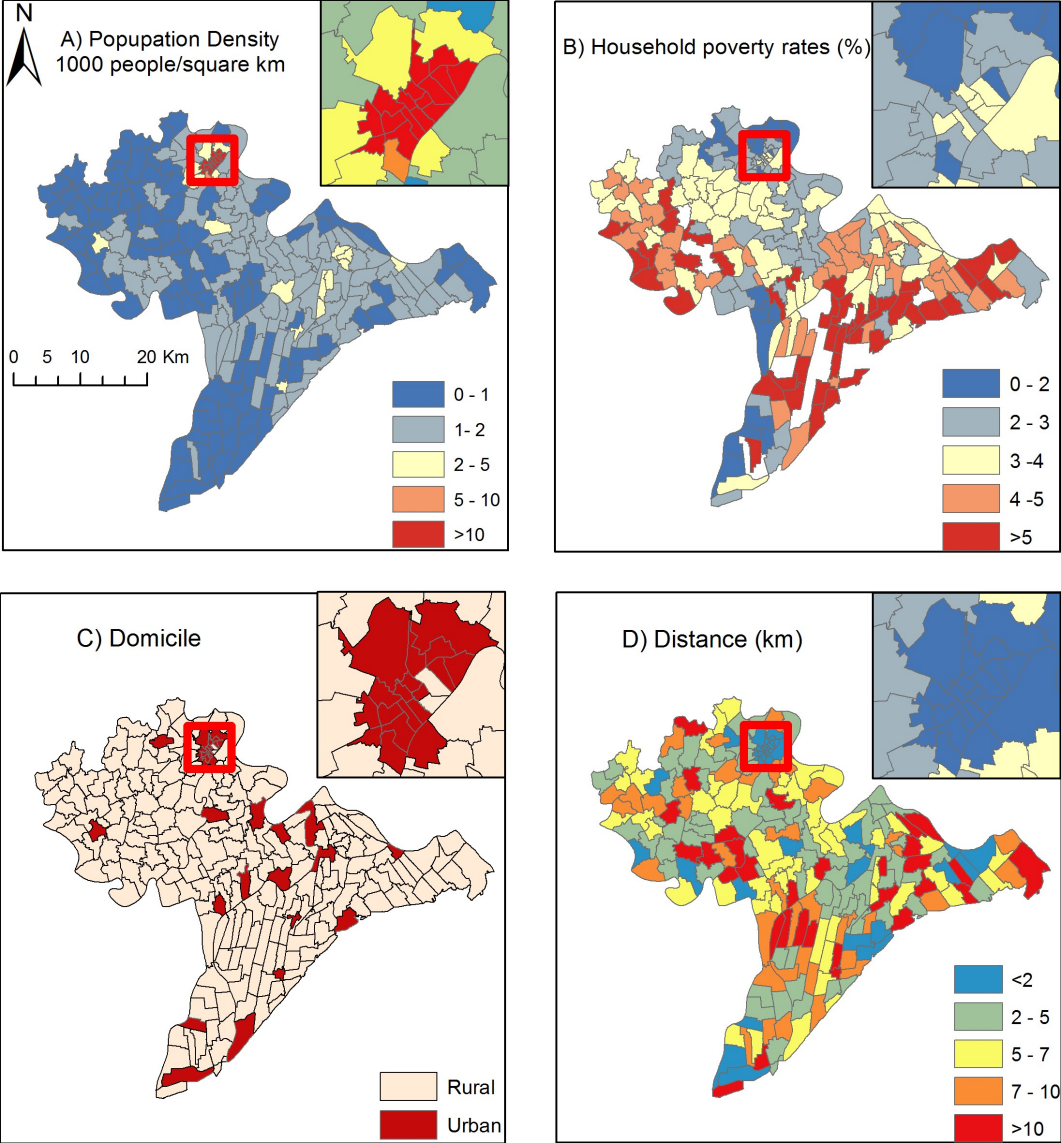
Spatial distribution of exploratory variables.

Table 2 and Fig 1 provide a summary and the geographical distribution of population density, household poverty rates and distances to the closest TB diagnostic units. As can be observed from Fig 1A and 1C, the urban population density was remarkably higher than that of the rural area. However, the household poverty rate followed a heterogeneous pattern across the study area (Fig 1B). However, most urban areas presented lower household poverty rates than rural areas. Likewise, distances to the closest TB diagnostic units are undoubtedly shorter for urban communes than for rural communes, as TB units are often found in urban areas of districts (Fig 1D).

**Table 2:** Summary of descriptive statistics of population density, household poverty rates and distance.

	Population density (1000 inhabitants/km^2^)	Household poverty rates (%)	Distance (km)
**Min**	0.51	0.85	0.24
**1**^**st**^ **quartile**	0.85	2.82	2.88
**Median**	1.10	3.78	5.13
**3**^**rd**^ **quartile**	1.41	5.17	7.70
**Max**	33.33	7.86	18.81

Table 3 shows that in the GLM Poisson regression model, the intercept and DIST are at a significance level of 1%. The DIST has a negative effect on TB notified incidence; when the distance from a commune to the nearest TB unit of the same district increased by 1 km, the notified TB incidence decreased by a factor of 0.87. The goodness-of-fit test showed that the model fits the data well. VIF exploratory variables showed that the GLM results are not biased by multicollinearity. However, residuals of the GLM Poisson model still exhibited positive spatial autocorrelations (global Moran’s I = 0.09, p <0.05). These results suggest that a GLM Poisson model cannot address unobserved spatial non-stationary relationships.

**Table 3:** Summary statistics of the GLM Poisson regression model.

Variable	Coefficient	Standard Error	t-value	p-value	Variance inflation factor
**Intercept**	0.37	0.1	3.7	<0.001	
**DEN**	-0.0008	0.0004	-0.20	0.85	1.59
**POOR**	0.02	0.2	1	0.33	1.17
**DOM(urban)**	0.12	0.08	1.5	0.14	1.52
**DIST**	-0.024	0.028	-0.88	<0.001	1.26

Corrected Aikake information criterion: 251.53

Residual deviance: 241.21. Goodness-of-fit test: p = 0.2, Degree of freedom: 224

Table 4 shows that the GWPR model with a spatially varying intercept and independent variables significantly lowers the AICc relative to the GLM model (249.52 and 251.52, respectively). As spatial heterogeneity is captured in the GWPR model, the residuals do not show spatial autocorrelations (global Moran’s I = 0.08, p = 0.07).

**Table 4:** Summary of the local GWPR model.

Variable	Minimum	Mean	Standard Deviation	Maximum
**Intercept**	0.30	0.32	0.02	0.35
**DEN**	-0.01	-0.002	0.006	0.017
**POOR**	0.03	0.03	0.02	0.05
**DOM(Urban)**	-0.02	0.04	0.01	0.07
**DIST**	-0.16	-0.14	0.02	-0.09

Bandwidth size: 229

Corrected Aikake information criterion (AICc): 249.52

Residual deviance: 233.41. Goodness-of-fit test: p = 0.2, Degree of freedom: 221

In Table 5 the GWPR shows slight but significant fit for the full local GWPR model with an AICc improvement of 3.81 whereas the adaptive bi-square bandwidth is reduced from 229 to 66. Spatial autocorrelation effects were also removed (global Moran’s I = 0.05, p = 0.2). All communes had positive intercept. Population density became the local (varying) variable while other exploratory variables remained as global (fixed) variables. The coefficients of population density observed were mostly negative values while those of other communes were positive but close to zero. The 95% CI of the coefficient of distance did not contain the value of zero, showing that distance has a significant effect on TB notified incidence. By contrast, the 95% CI of the coefficient of the household poverty rate and urban areas did not differ from zero, suggesting that the effect of the two variables on response variables is not statistically significant.

**Table 5:** Summary of semiparametric GWPR model.

Local variable	Minimum	Mean	Standard Deviation	Maximum
**DEN**	-0.84	-0.27	0.006	0.11
**Global Variable**	**Value**	**Standard** **Error**	**t-value**	**95% Confidence Interval**
**Intercept**	0.26	0.06	4.33	(0.14;0.38)
**POOR**	0.004	0.030	0.016	(-0.055;0.063)
**DOM(Urban)**	-0.02	0.04	0.23	(-0.10;0.06)
**DIST**	-0.14	0.03	-4.2	(-0.20;-0.08)

Bandwidth size: 66

Corrected Aikake information criterion: 245.71, Residual deviance: 221.12

## Discussion

We used GIS to visualize the geographic distributions of TB notified incidence in relation to social factors such as population density, household poverty rates and proximity to the nearest diagnostic services in the same district. Local and semiparametric GWPR models were compared to the conventional GLM Poisson model to find the best fitting model to investigate the effect of social factors and distances on TB notified incidence.Obviously, the calibration of GWPR models shows clear improvements in model fit relative to the global Poisson regression model.

TB notified incidence marginally increased from 79 to 84 per 100,000 population while the number of TB diagnostic services did not increase. However the overall population of the province of Nam Dinh slightly increased during the study period (2% annually on average). This finding shows that the NTP, which mainly relies on passive case findings, was successful at maintaining the coverage of basic TB diagnostic services.

According to the results of the semiparametric GWPR model, distances to TB diagnostic facilities have significant negative effect on TB notified incidence after adjusting for household poverty rates, population density and urban domiciles. This may be the case because communes positioned farther away from TB diagnostic facilities enjoy less or no access to such services, thus hindering case detection. While TB units are located in urban areas, the fewer TB cases detected in urban communes imply that TB cases are detected in rural communes surrounding urban communes. Evidence suggested that distance is recognized as an important barrier to health service access [35]. A study conducted in Ethiopia also showed that when the distance from the nearest TB diagnostic unit increases by 1 km, the notification rate of smear-positive pulmonary TB decreases by 0.25 per 100,000 inhabitants. Several studies showed that a longer distance to TB services may lead to delays in the delivery of initial healthcare consultations [36–38]. Population density was found to spatially vary across the province, suggesting that the association between TB incidence and population density could be masked by latent differences in socioeconomic status and lifestyle of geographic areas. Previous studies suggested that TB incidence is likely correlated with numerous biological and socioeconomic factors and especially with sanitation, HIV prevalence, child mortality, and smoking and diabetes rates [27]. Moreover, TB incidence should likely depend on the actual burdens of TB and on the effectiveness of TB case detection activities. These factors distribute unevenly across geographic regions and are difficult to confine. Therefore, this topic calls for further investigations.

GIS serves as an effective tool for TB programming in visually investigating trends of TB incidence and its relations to social and spatial factors. In combination with GIS, GWPR techniques, which allow regression parameters to vary spatially, exhibit superior performance when applied to the global GLM Poisson model in examining non-stationary effects of social and spatial predictors on TB incidence. Given the advantages of using GIS for TB programming, we strongly suggest that GIS programs be applied to TB surveillance systems to converge TB-specific data with more detailed social and demographic features and variables related to the performance of TB programs. These data would be useful in calibrating predictive models and in offering policy makers insight into the spatial patterns of TB and its determinants and therefore, plan locally adaptive interventions, improving the effectiveness and efficacy of Vietnam NTP.

This study presents several limitations. First, as the analysis was based on the existing data of other institutions, we could not control the data collection process or the validation of measurements. Second, as data were analyzed at the communal level, relationships found between geographic distance and TB notified incidence cannot be inferred to an individual level. Third, this study was also sensitive to “scale effects”, an aspect of the Modifiable Areal Unit Problem that can change results when data are aggregated by geographic boundaries of different levels. Forth, the Euclidean distance used for the analysis is not an actual travel distance. Travel distances and lengths of time patients need to travel from residential locations to TB facilities could differ between urban and rural areas and between different topographical and transportation scenarios. Nevertheless, our study can contribute to the literature on geographic distance and on its relationship to TB case detection and may stimulate further research on this topic.

To the best of our knowledge, this is the first study to combine TB surveillance, demographic and socioeconomic data in GIS and to use GWPR to analyze the geographic distances to TB diagnostic services and other social risk factors and their relationships with TB incidence in Vietnam. Our findings could assist policy makers at the provincial level in mobilizing resources and in expanding the NTP to provide proper diagnostic services by improving transportation systems, opening additional clinics or initiating outreach to remote areas.

## Conclusions

GIS technologies benefit TB surveillance system as a tool to scrutinize the association of TB-specific and sociodemographic characteristics of population. Distances to closest TB diagnostic facilities were found to be a major factor influencing TB notified incidence. Hence, distances should be considered when planning actions to improve access to those who live far from TB diagnostic services, thereby improving TB detection. Additional studies must confirm the association between geographic distance and TB case detection and must explore other factors that may affect TB notified incidence.

## Supporting information

S1 Fig Spatial distribution of TB cases and the local Moran’s I. (TIF)
