# Supplementary figures and images for: Use of Geographically Weighted Poisson Regression to examine the effect of distance on Tuberculosis incidence: A case study in Nam Dinh, Vietnam
Source: PLoS One. 2018 Nov 12;13(11):e0207068. doi: 10.1371/journal.pone.0207068 (PMC6231628; doi:10.1371/journal.pone.0207068)

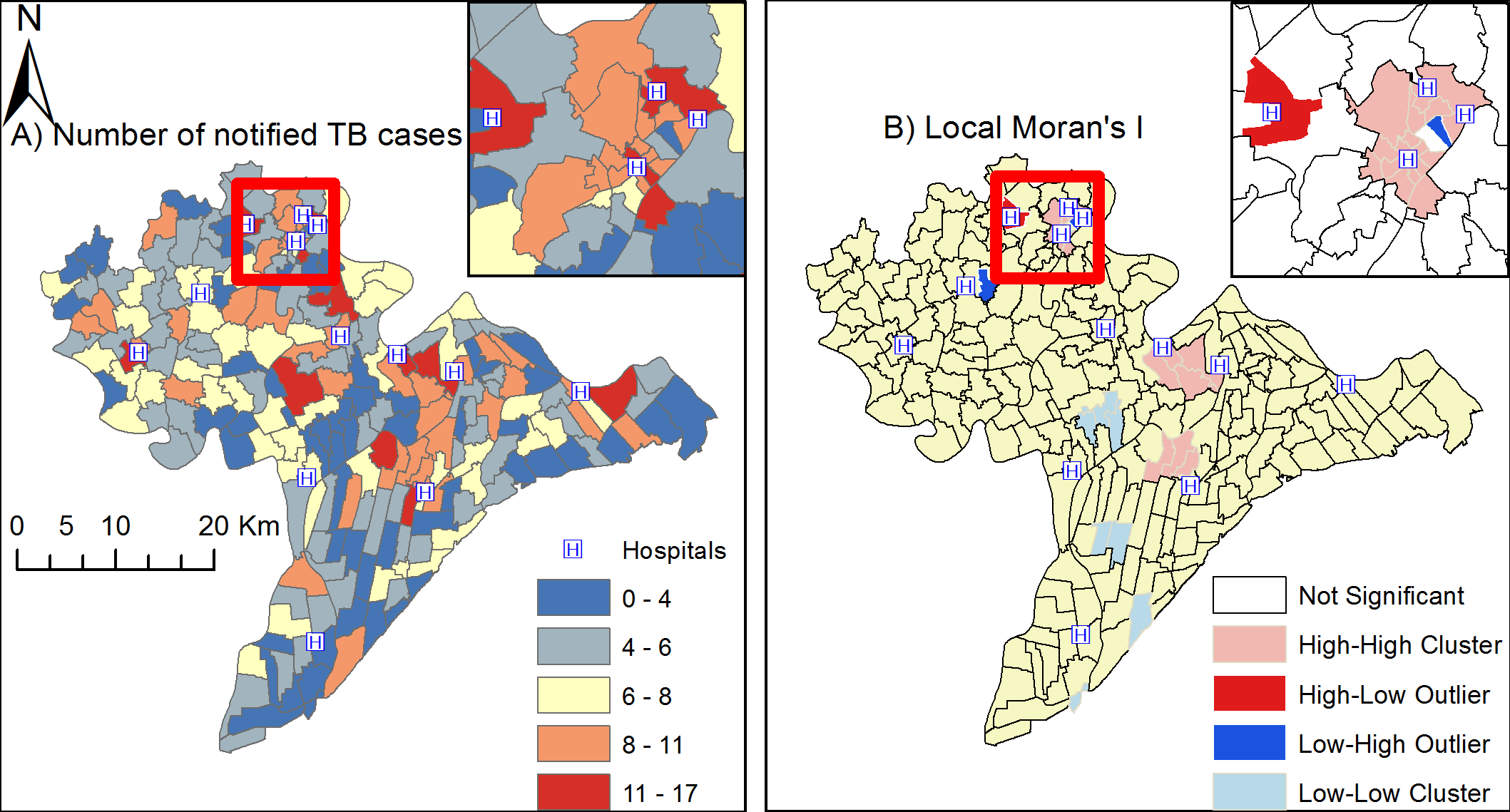

Supplement: S1 Fig — (TIF) [file pone.0207068.s001.tif]
